# Thymic Epithelial Tumor with Heart Metastasis in a Horse

**DOI:** 10.4061/2010/386378

**Published:** 2010-08-08

**Authors:** Farshid Shahriar, Janet Moore

**Affiliations:** California Animal Health and Food Safety Laboratory, San Bernardino Branch, University of California, Davis, 105 West Central Avenue, San Bernardino, CA 93408, USA

## Abstract

Thymic malignancy is rare in horses. Thymic epithelial tumor was diagnosed in an 18-year-old mare with invasion and metastasis to the pericardium and heart. At necropsy, the cranial thoracic cavity was obliterated by a large mass located in the thymic region and the right atrium was also expanded and effaced by a similar mass. Histologically, the neoplasm was composed of sheets of spindle cells with intraparenchymal Hassall's corpuscles and formation of pseudorosettes around blood vessels compatible with type A thymic epithelial tumor according to World Health Organization classification. The neoplastic cells were diffusely immunoreactive for cytokeratin and negative for vimentin, S100, neuron specific enolase, glial fibrillar acidic protein, chromogranin A, synaptophysin, CD3 and CD79a markers. To the authors' knowledge, cardiac invasion and distinct histological pattern of pseudorosette formation have not been described in equine thymic epithelial tumors previously.

## 1. Case Report

The thymus is a lymphoepithelial organ that originates embryologically from the third and fourth pharyngeal pouches and undergoes gradual involution as animals mature [1]. 

Thymoma, a neoplasm of thymic epithelial cells, is reported uncommonly in a variety of domesticated animals [2–5] and rarely in the horse [6–8]. Although thymomas appear benign histologically, clinically they are characterized as benign or malignant, according to their invasive behaviour. Described here is a case of thymic epithelial neoplasm in a horse with pericardial invasion and right heart metastasis. 

An 18-year-old, Tennessee Walking Horse mare was submitted for necropsy to the San Bernardino branch of the California Animal Health and Food Safety Laboratory system, with a history of dyspnea and death during an uphill trail ride. 

**Figure 1:**
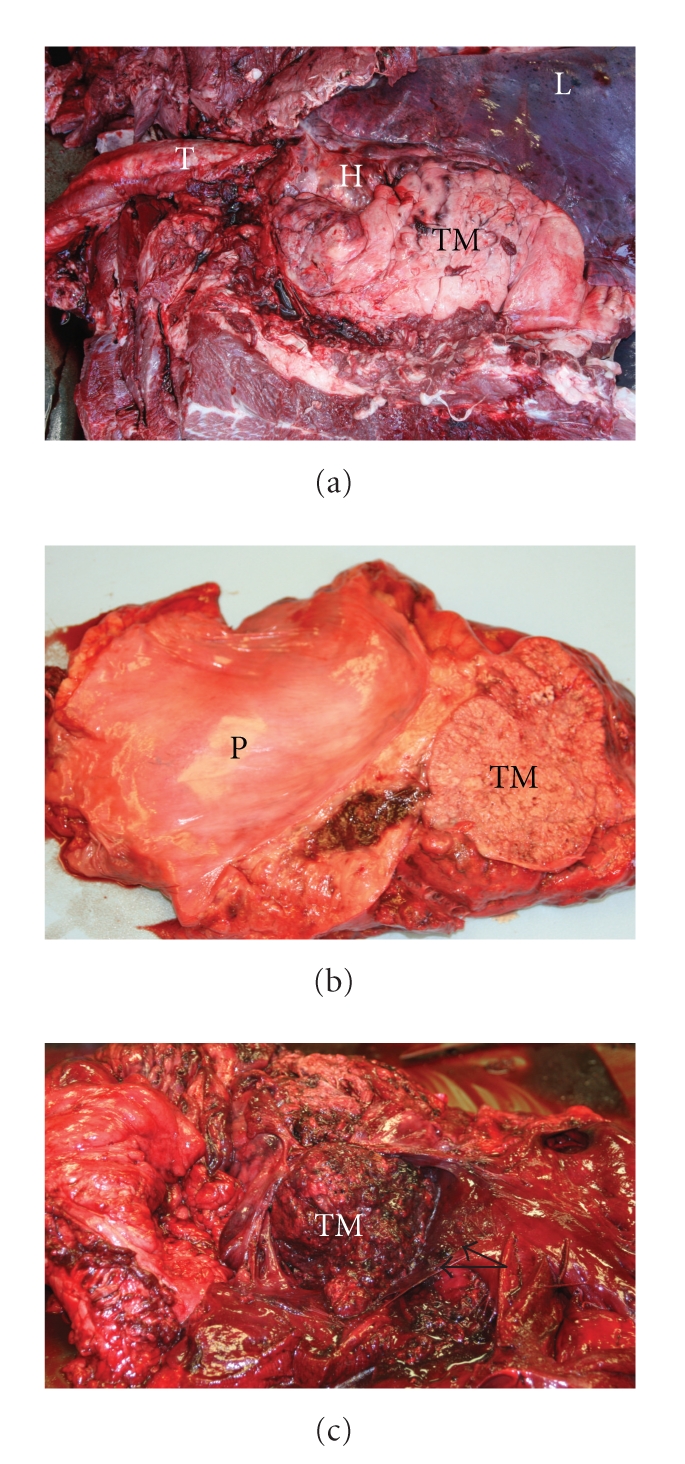
Thymic mass with heart invasion. (a) Thoracic cavity. The cranial thoracic cavity is occupied by the thymic mass (TM) compressing the heart (H). The mass is adhered to cranioventral thoracic wall, and left side of the pericardial sac. L: Lung, T: trachea. (b) A section of pericardial sac (P) with adjacent thymic mass (TM). The thymic mass is attached and invading the pericardial sac. (c) Dissected right atrium and atrioventricular valves. The right atrium is markedly expanded and effaced by the metastatic thymic mass (TM) obliterating the right atrioventricular valves (arrows).

At necropsy, the pleural cavity and pericardial sac contained approximately 500 and 60 ml of serosanguineous fluid, respectively. The cranial thoracic cavity was occupied by a large (approximately 35 × 25 × 20 cm) firm, irregular, multilobulated, white-tan mass (Figure 1(a)). There was extensive fibrous adhesion between the mass and both the cranioventral thoracic wall, and left side of the pericardial sac (Figures 1(a) and 1(b)). The right atrium was markedly expanded and effaced by a firm, poorly defined mass that obliterated the right atrioventricular valve (Figure 1(c)). The lungs were diffusely congested and edematous, and the liver was slightly firm with a prominent acinar pattern. 

**Figure 2:**
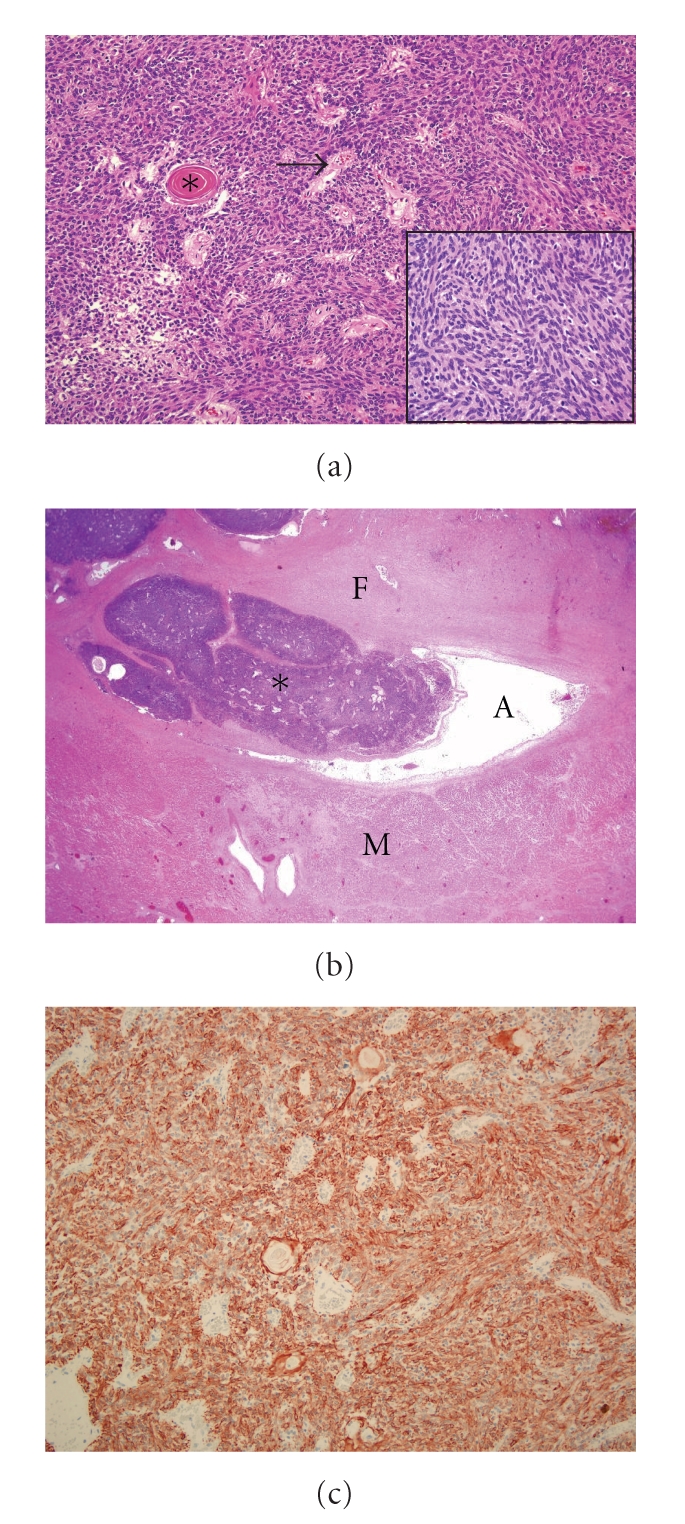
Photomicrographs of the thymic mass with heart metastasis. (a) The neoplastic cells in thymic mass form solid sheets. Scattered Hassall's corpuscles (*) present in the mass. Pseudorosette formation is evident around blood vessels (arrow) with a tendency to form a palisading basal cell layer. Haematoxylin and eosin. Magnification x100. Inset: Higher magnification of the thymic neoplastic cells with spindle/oval shape nuclei. Magnification ×600. (b) Right atrial wall. The metastatic thymic neoplasm (*) invading the right atrial wall. M: Myocardium. F: adventitial connective tissue of entering vessel into the right atrium. A: part of the right atrial chamber. Haematoxylin and eosin. Magnification x20. (c) Thymic neoplasm; cytokeratin immunohistochemical staining of the neoplastic cells. The neoplastic cells have positive cytoplasmic immunohistochemical labeling for pancytokeratin. Avidin-biotin immunoperoxidase—Amino-Ethyl-Carbazol chromogen. Magnification x100.

Tissue samples from the thoracic and cardiac masses, heart, lung, liver, spleen, urogenital and gastrointestinal tracts, thyroid and adrenal glands, brain, and skeletal muscle were collected and fixed in 10% neutral buffered formalin, embedded in paraffin, sectioned in 4 *μ*m, and stained with hematoxylin and eosin for histologic examination. The thoracic mass was unencapsulated, well demarcated, and composed of variably sized lobules of neoplastic cells separated by thick fibrous connective tissue trabeculae. Within the lobules, solid sheets of neoplastic cells were supported by scant fibrovascular stroma, and scattered pseudorosettes were formed by perivascular palisading of neoplastic cells (Figure 2(a)). The tumor cells were spindle-shaped with indistinct cell borders, small to moderate amounts of eosinophilic cytoplasm, and a large ovoid to elongated nucleus with coarse granular chromatin pattern and absence of nucleoli (Figure 2(a)—inset). The mitotic rate was 0-1 per high power field (40x). The parenchyma of the mass contained randomly scattered Hassall's corpuscles, characterized by concentric whorls of keratinizing epithelial-reticular cells (Figure 2(a)), and occasional small foci of lymphoid cells. 

Histologic examination of the right atrial wall revealed a large unencapsulated, multinodular infiltrative mass of monomorphic neoplastic cells with similar cytologic features to those of the thoracic mass invading the right atrial wall, lumen, and proximal right ventricular wall (Figure 2(b)). 

Within the liver, diffuse bridging periacinar fibrosis and frequent severe, centrilobular hemorrhagic necrosis, were compatible with chronic passive congestion. The lungs were diffusely congested and edematous with multifocal, acute intra-alveolar hemorrhage. The results of routine bacterial cultures and mineral analysis of the liver were unremarkable. 

Selected sections of both tumors were stained for: pancytokeratin (cytokine AE1/AE3), vimentin, S100, neuron specific enolase, glial fibrillar acidic protein, chromogranin A, synaptophysin, CD3, and CD79a immunohistochemical markers using an avidin-biotin technique. The cytoplasm of most neoplastic cells was diffusely positive for pancytokeratin (Figure 2(c)). All such cells were negative for vimentin, all neuroendocrine markers, CD3, and CD79a. 

Previously thymomas were divided into three histologic types: lymphocyte predominant, epithelial predominant, and mixed [3]. However, in the recent commonly used classification of thymic epithelial neoplasms by the World Health Organization (WHO) [9] and later modification [10] the thymomas are typed into A, AB, B1, B2, and B3 in which type A thymoma is characterized by proliferation of spindle-shaped cells with oval to elongated nuclei, lacking nuclear atypia containing few or nonneoplastic lymphocytes and inconspicuous nuclei [9]. Type A thymoma can have a range of unusual morphologic appearances including pseudorosette growth pattern with radial arrangement of neoplasitc cells around small blood vessels [10]. The presented case has characteristics of type A thymic epithelial tumor with spindle-shaped epithelial cells and pseudorosette formation around vessels. Hassll corpuscle formations had been described in type A thymomas [9] as we see it in our case as well. All thymic epithelial tumors (type A and AB) can show malignant and aggressive behavior [11, 12]. 

Thymic epithelial tumor is rarely reported in horses [6–8] and most other animal species. Goats are the exception with a significantly higher reported incidence [2, 5, 13]. Of the limited number of reported equine thymic epithelial neoplasms, two cases in Japan were behaviorally benign with no metastases, [7] and only two cases of thymic carcinoma were reported in horses with metastases to lung, thyroid, rib, kidney and lymph nodes [6, 8]. 

In this case, the thymic tumor had metastasised to the heart, a site not previously reported in horses. Both the primary thymic and metastatic cardiac tumors had a solid pattern of neoplastic cells with pseudorosette formation. An angiocentric distribution has been described in some thymomas [3] and such growth pattern was associated with type A thymic epithelial tumors [10]. However it has not been reported previously in cases of equine thymic neoplasms. No eosinophils were noted in this thymic mass, unlike reports of thymoma in other species [5].

It has been suggested that thymoma may metastasise via blood and lymph vessels [6]. In this case, the exact method of cardiac invasion was not determined; however, metastasis via the lymphatic vasculature to the right heart, or by direct invasion, would seem likely. 

Differential diagnoses for the mass reported in this article include anaplastic bronchogenic carcinoma and aortic body tumors. Bronchogenic carcinomas may present as a solitary mass at the bifurcation of the trachea, or multiple masses throughout the lung [14, 15]. The mass in this case was separate from the trachea and situated where the thymus is normally located, and the presence of Hassall's corpuscles within the primary mass and metastasis was considered diagnostic of thymoma. Aortic body tumors have been described rarely in horses and have differentiating neuroendocrine, histologic, and immunohistochemical features [16] including negativity for cytokeratin [17], a marker for which the neoplastic cells in this case were strongly positive. 

This report describes a rare thymic epithelial tumor in a horse. The anatomic location and histologic appearance of this tumor fulfill the definitive diagnostic features of a type A thymic epithelial thymoma.
